# Coastal pollution from the industrial park Quintero bay of central Chile: Effects on abundance, morphology, and development of the kelp *Lessonia spicata* (Phaeophyceae)

**DOI:** 10.1371/journal.pone.0240581

**Published:** 2020-10-15

**Authors:** Carolina Oyarzo-Miranda, Nicolás Latorre, Andrés Meynard, Jorge Rivas, Cristian Bulboa, Loretto Contreras-Porcia

**Affiliations:** 1 Departamento de Ecología y Biodiversidad, Facultad de Ciencias de la Vida, Universidad Andres Bello, Santiago, Chile; 2 Centro de Investigación Marina Quintay (CIMARQ), Facultad de Ciencias de la Vida, Universidad Andres Bello, Santiago, Chile; 3 Center of Applied Ecology and Sustainability (CAPES), Santiago, Chile; 4 Programa de Doctorado Medicina de la Conservación, Facultad de Ciencias de la Vida, Universidad Andres Bello, Santiago, Chile; Kaohsiung Medical University, TAIWAN

## Abstract

The industrial park of Quintero Bay (QB) in the central coast of Chile was established in the 1960s, presents high levels of pollution due to the industrial activity, and it is known as one of the five Chilean “sacrifice zones”. *Lessonia spicata* is the most important habitat-forming kelp species in the intertidal along the central and south shores of Chile, and currently there are no morphometric and population studies of *L*. *spicata* (or other seaweed species) nor studies about the effects of pollution on its development in QB and neighbouring sites. In this context, the aims of this study were (i) to register the abundance and morphological features of *L*. *spicata* populations from Ventanas, Horcón and Cachagua (sites with different pollution histories and located only up to 40 km from the QB); ii) to determine the heavy metals (HMs) concentration in seawater and marine sediments; and (iii) to evaluate *in vitro* the effects of exposure to seawater from the three sampling sites on spore release and early developmental stages, up to the juvenile sporophyte. Results showed that the chronically exposed Ventanas kelp population had the smallest adult individuals in comparison with the other sites. Ventanas and Horcón registered high HMs concentration in the seawater and marine sediments exceeding the international permissible limits (e.g in seawater Cu 20–859 μg L^-1^; sediments Cu > 50,000 μg kg^-1^). Unexpectedly in Cachagua, a site often considered unpolluted, high concentrations of Cu and As were also registered in the seawater (859 and 1,484 μg L^-1^, respectively) and of As in marine sediments (20,895 μg kg^-1^). Exposure of gametophytes to the seawater from Ventanas resulted in a developmental delay compared to the other treatments; however, low sporophyte production was determined in all treatments. Our results indicate that QB, more notably Ventanas, induce highly negative effects on individual development, and consequently on seaweed populations, which suggest a long-term negative impact on the community structure of these marine zones. Furthermore, the high concentrations of HMs reported here at Cachagua suggest a recent expansion of pollution along the central coast of Chile, evidencing effects on the marine ecosystem health even on sites far from the pollution source.

## 1. Introduction

Heavy metals (HMs) are inorganic pollutants naturally present in the environment, which have increased significantly due to anthropogenic action [1]. These metals generate alterations in the normal metabolism, development, and fitness of animals, plants and seaweeds, depending on their concentration, distribution and speciation [2–4]. Several reports have evidenced the direct impact of heavy metal pollution on loss of biodiversity in coastal ecosystems [5–8].

Seaweeds are bioindicators and biomonitors of heavy metal enrichment [9, 10], especially brown seaweeds (Phaeophyceae), which are capable of adsorbing more heavy metals than other species because of the alginate and fucoidan matrix present in their inner cell membrane [11, 12]. Species of Phaeophyceae, such as *Scytosiphon lomentaria*, *Macrocystis pyrifera* and *Ectocarpus siliculosus*, are highly tolerant to Cu excess [13–15]. In contrast, species of the genus *Undaria* and *Lessonia*, are sensitive to high concentrations of HMs, principally Cu, which trigger oxidative stress, cell damage and negative effects on spore release, germination and gametophyte development [4, 16–20]. Recently, Contreras-Porcia et al. 2020 [17] demonstrated that binary and tertiary mixtures of Cu, Cd, and polycyclic aromatic hydrocarbons (PAHs) have negative synergistic effects on spore settlement of the kelps *Lessonia spicata* and *M*. *pyrifera*.

The life cycle of kelps (order Laminariales) consists of two phases, a microscopic haploid gametophyte (*n*), alternating with a macroscopic diploid sporophyte (*2n*), which respond differently to environmental variables [21]. Specifically, the spores and gametophytes are more sensitive to chemical pollution exposure than the adult sporophytes [17, 22]. Moreover, microscopic stages play a critical role in the persistence and sustainability of kelp populations, and therefore, harmful effects of pollution may reduce or cause local extinction [23, 24]. Indeed, the adult sporophyte populations are an indicator of the pollution levels because as soon as recruitment occurs, they are immediately subjected to run-off and other pollutants present in the seawater and marine sediments [25]. For example, sporophytes of *Laminaria japonica* exposed to chronic pollution (East Japanese Sea) show anatomic alterations, inhibition of the generative tissue development, and a delay in maturation of the sorus [26]. The negative effects on any of the life stages, produce direct and indirect economic, biological, and ecological effects, which have repercussions on higher levels of biological organization, such as population, community, and ecosystem [7, 27, 28].

Along the coast of Chile are located several industrial parks that concentrate energy production companies, fuel refineries and others, which have a long history of pollution problems, affecting terrestrial and coastal zones around the industrial area [29–31]. One of them is the industrial area of Puchuncaví-Ventanas (32° 44´S 71°30´W), located in Quintero Bay (QB) at the central coast of Chile. This industrial park began operation in 1961, and it is characterized by high pollution levels due to historical discharges of petroleum, gaseous pollutants and atmospheric particulates, as well as the deposition of heavy metals from diverse industrial facilities, including coal-fired power plants, a copper refinery and smelter, natural gas terminals, and cement companies, among others [30–35]. Since the industrial activities in the zone were largely unrestrained and their impacts were poorly regulated during several decades, the QB is known nowadays as one of the five Chilean sacrifice zones [31]. Previous studies about marine pollution in this zone reported historical and current concentrations of HMs such as Al, Mo, Fe, Cr, Cu, and Zn, and PAHs that exceed the permissible limits of the US Environmental Protection Agency (US EPA) quality guidelines for seawater, and were higher than quality standards allowed in molluscs, crustaceans and kelps for human consumption [30, 36].

It is worth highlighting that at present there is no information about the status of kelps forest in the Quintero Bay, such as *L*. *spicata*, despite their key economic and ecological role. *L*. *spicata* is an important ecosystem engineer in the low intertidal zone along the central and south coasts of Chile [37]. This kelp facilitates the recruitment of algae and invertebrates and provides habitat and shelter for several associated species in its holdfast and blades, modulating local biodiversity and community structure [38]. In addition, intertidal beds of *L*. *spicata* are an important commercial resource exploited by seaweed-based industries for alginate, and animal consumption [39, 40]. Based on this previous knowledge, we formulated two hypotheses: (i) *L*. *spicata* populations chronically exposed to seawater from polluted sites that are closer to QB would be more affected in terms of abundance and morphometric features than more distant kelps populations, and (ii) the *in vitro* exposure of *L*. *spicata* to seawater from polluted sites closer to QB would have a higher negative effects on spore release and early developmental stages than the exposure to seawater from more distant sites. In this context, the aims of this study were (i) to register the abundance and morphological features of *L*. *spicata* populations from Ventanas, Horcón and Cachagua (sites with different pollution history and located only up to 40 km from the QB), ii) to determine the heavy metals (HMs) concentration in seawater and marine sediments, and (iii) to evaluate *in vitro* the effects of exposure to seawater from the three sampling sites on spore release and early developmental stages of this kelp.

## 2. Materials and methods

### 2.1. *In situ* analysis

#### 2.1.1. Sampling sites

We defined the industrial park located in Quintero Bay (32°44′31″ S, 71°29′33″ W) as a pollution focus. Then, we selected three sampling sites, due to their order of proximity to the focus, from south to north: Ventanas, Horcón and Cachagua (S1 Fig). Ventanas (32°44′31″ S, 71°29′33″ W) and Horcón (32°42′33″ S, 71°29′19″ W) where closer to QB and had serious environmental precedents [30, 41, 42], while Cachagua (32°35′04″ S, 71°27′22″ W) was the farthest site from QB. This site was declared as a natural sanctuary in 1979 and does not have a history of direct anthropogenic impact [13, 43, 44]. The specific sampling sites were outside of the delimited protected area, and only part of the reproductive tissue (1–2 cm^2^) was collected, where extraction permits are not required [45].

#### 2.1.2. Material treatment

For the analysis of total HMs concentration, all glass and plastic materials (flasks, plastic bottles, Petri dishes, glass pipettes, among others) were treated according to the protocol established by the US EPA [46]. The material was incubated on the non-ionic detergent Extran 7% (Merck, Germany) for 12 h. Then, the material was rinsed three times with distilled water, incubated on nitric acid 5% (Merck, Germany) for 12 h. After incubation, the material was washed with distilled water and MilliQ water (18,2 MΩ cm) three times. Finally, it was dried on a Class II laminar flow chamber (Cientec, Model JSCB-1200SB), avoiding any contact with metal.

#### 2.1.3. Abundance and morphometric features

**Fig 1:**
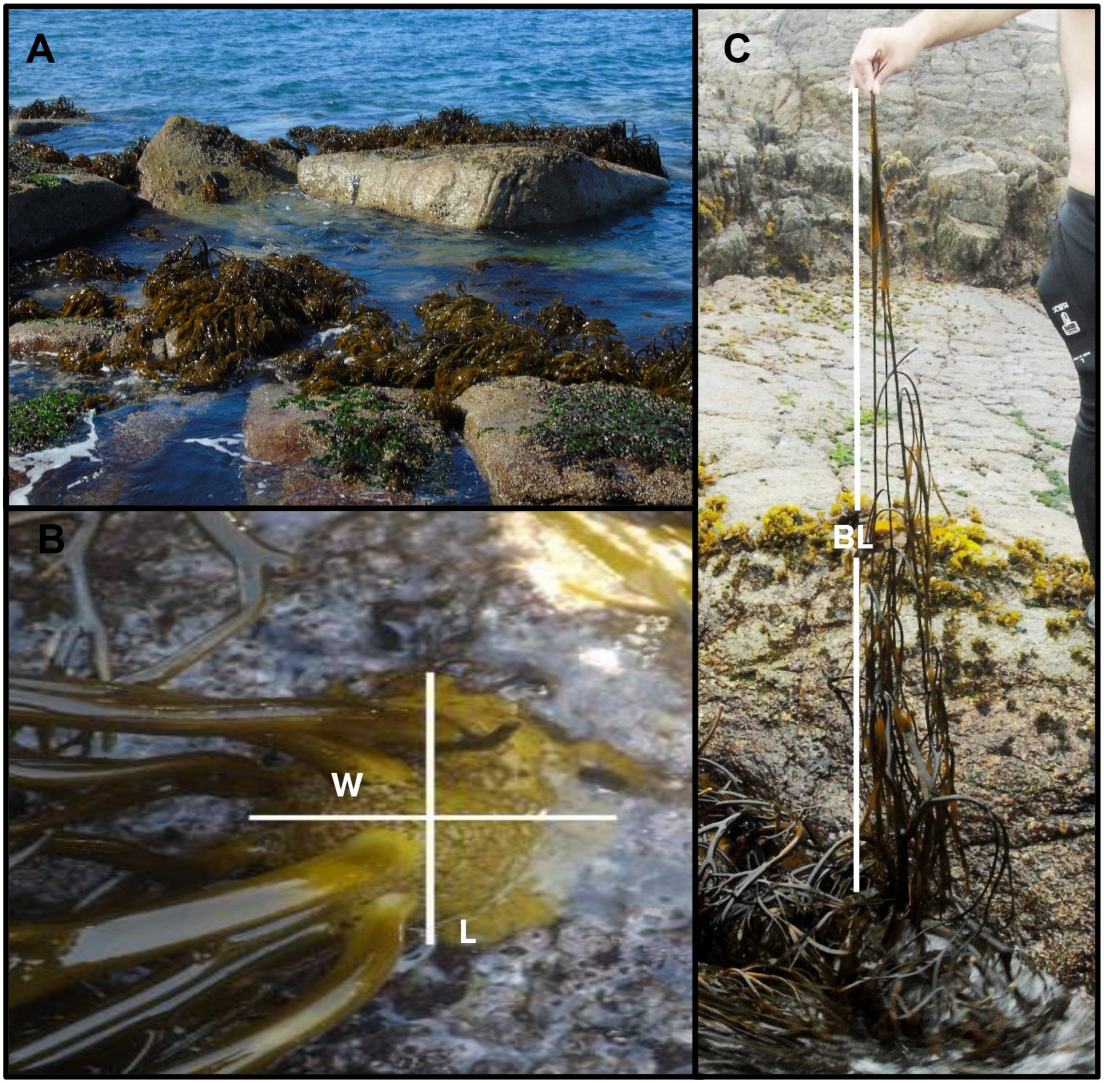
Morphological features recording. A) *Lessonia spicata* intertidal populations examined at Cachagua sampling site, B) view of a holdfast of *L*. *spicata* showing its length (L) and width (W), and C) illustration of the measurement of the longest blade (BL) in an individual of *L*. *spicata*.

The study was carried out in July (2018), because it corresponds to the season with the highest fertility of *L*. *spicata*, as reported previously by Araujo and Faugeron [47]. For the three sites, we randomly established four areas of 2.25 m^2^ at the low intertidal zone where populations of *L*. *spicata* occur. In each area, we quantified the number of holdfasts, the length of the longest blade and the diameter of the width and length of each holdfast (Fig 1). Based on Vega et al. [27], the diameter of the holdfast was used to classify the size structure of *L*. *spicata* population: recruits and juvenile (< 10 cm), young (10–19 cm) and adult sporophytes (> 20 cm). Additionally, the holdfast area was calculated using the following equation:

Holdfastarea=π*diameter(width*length)

#### 2.1.4. Collection and analysis of total concentration of heavy metals in seawater and marine sediments

Seawater and marine sediments samples from Ventanas, Horcón and Cachagua were collected manually in an area close to the *L*. *spicata* population (3 m perimeter), in triplicate for each matrix. For seawater and sediment samplings, the material was rinsed with seawater from each site three times. Samples of seawater were taken using 500 mL acid-washed low-density polyethylene bottles, which were placed in plastic bags. In the case of marine sediment, around 50 cm of surface sediment was collected using 50 mL Falcon tubes. Both types of samples were placed in a cooler (treated for HMs) with ice packs and kept at 8–10°C for transporting. Once at the laboratory of Quintay Marine Research Center (CIMARQ) (33°11′34.567″ S, 71°42′8.842″ W), sediment samples were placed on plastic Petri dishes and oven-dried for 12 h at 45°-50°C. All samples were sent to the Laboratory of Elemental Analysis (LAE) at the Universidad Andrés Bello, Concepción. We selected Cu, As, Cd, Ag, and Pb because of their toxicity in marine organisms and high levels in QB according to previous reports [34, 43, 48, 49]. The analyses of the total concentration of HMs were carried out through inductively coupled plasma-mass spectrometry (ICP-MS Aurora M90 model Bruker, USA).

EPA3051/3050 method [50] was used for sample treatments.

Because Chile lacks quality guidelines for seawater and marine sediments, the results of the HMs concentrations were contrasted with international criteria. In the case of seawater samples, they were compared with the values established by the US EPA standards in the "National Recommended Water Quality Criteria" [51]. These standards compare two criteria according to the exposure time (acute and/or chronic), where exceeding these values indicates possible damage to marine organisms. On the other hand, sediments samples were compared with the values established by the "Canadian Sediment Quality Guidelines for the Protection of Aquatic Life" [52, 53], which applies two values: threshold effect level (TEL) indicates a criterion that damage to marine organisms may occur if this value is exceeded; and possible effect level (PEL) corresponds to the lowest value associated with adverse effects in marine organisms.

### 2.2. *In vitro* experiments

#### 2.2.1. Treatments

We collected 10 L of seawater from each sampling site, and they were stored in high-density pre-treated polystyrene containers. Then, seawater was filtered through 0.22 μm pore membrane (Sartorius, Goettingen, Germany) and stored in new glass bottles in darkness without any enriched culture medium at 15°C during all the experimental time (two months).

#### 2.2.2. Kelp sampling for *in vitro* experiments

The biological material was collected from Cachagua due to its low polluted environmental history. Blades with sori, were collected randomly in an area of 10 m^2^. Then, the sori were cleaned to eliminate epibionts using the following sequence: washing with iodine at 9% dissolved in 0.22 μm filtered-seawater, rinsed with distilled water, cleaned superficially with ethanol at 5% using a paint brush, rinsed with 0.22 μm filtered seawater plus GeO_2_ at 4% m/v, and finally rinsed with 0.22 μm filtered-seawater. Then, they were dried with absorbent paper, and transported in a cooler at 10°C to the Laboratory of Ecology and Molecular Biology in Algae (LEBMA, www.lebma.cl) in Santiago. We used the study of Contreras et al. [18] as a reference to classify the developmental stages: Spore (S), Spore with germ tube (G), Undifferentiated gametophyte (U), Female gametophyte (FG) and Male gametophyte (MG) (S2 Fig). The summary of the *in vitro* experiments was schematized in S3 Fig.

#### 2.2.3. Effect of treatment on spore release

Individual discs of 1 cm in diameter were cut from randomly selected sori. Each disc was blot-dried with absorbent paper, air dried on trays and exposed to light for 1 h to stimulate the spore release. Then, groups of four discs were placed in pre-treated amber glass vials of 5 mL containing 4 mL of treatment. The glass vials were kept under continuous agitation at 120 rpm on an orbital shaker (SEA STAR^™^), under controlled conditions (15°C and 20–30 μmol photons m^-2^ s^-1^) during the entire assay. The number of spores was recorded after 1, 2, 3, 5 and 7 hours, by counting three samples in a haemocytometer (Neubauer 0.100 mm, 0.0025 mm^2^) using a light microscope (Leica ICC50 HD, Germany).

#### 2.2.4. Effect of treatment on spore settlement

After three hours of spore release, 58 μL of the spore solution (with an average density of 5 x 10³ cells mL^-1^) were inoculated in glass Petri dishes containing 7 mL of treatment (n = 15). The count of settled spores was performed using an inverted microscope (Eclipse Ts2, Nikon, Japan) in ten areas of 1 mm^2^ per Petri dish at 48 h, and after the first medium change at 120 h. The culture conditions for all experiments were: photoperiod of 12:12, 10–15°C, and 20–30 μmol photons m^-2^ s^-1^.

#### 2.2.5. Effect of treatment on gametophyte development

The same cultures used for the spore settlement experiment (2.8) were maintained and monitored for the development of gametophytes. The development of gametophyte was recorded by counting the number of cells per stage (S, G and U) 6 times during 40 days of exposure. A total of 10 areas of 1 mm^2^ were evaluated per replicate (n = 15). The 7 mL of treatment were changed every seven days.

#### 2.2.6 Effect of treatment on sexual differentiation, sporophyte production and fertility

New cultures were conducted for sexual differentiation, sporophyte production and fertility experiments. For that, we added 65 μL of spore solution (5 x 10^3^ cell mL^-1^) in each Petri dish containing 7 mL of 0.22 μm filtered-seawater obtained from CIMARQ. After 25 days, the seawater was changed for the different treatments, data was recorded 3 times during 25 days of exposure, which was changed every seven days. Petri dishes were maintained at the same culture conditions mentioned on point 2.9. We evaluated a total of 7 areas of 1 mm^2^ per Petri dish (n = 13).

Cell numbers per stage (U, MG and FG) for sexual differentiation, and sporophyte numbers for sporophyte production were quantified, and then fertility was determined. Percentage of fertility was calculated according to Oppliger et al. [54], using the following equation:

$$\%Fertility=\frac{NumberofSporophytes}{Numberoffemalegametophyte+NumberofSporohpytes}(x100)$$

### 2.3. Data analyses

#### 2.3.1. Abundance and morphometric features

R software was used to perform all statistical analyses. We first checked for normality and homoscedasticity of the data by performing a Shapiro Wilk test and a Bartlett test, respectively. Based on these results, we performed a Kruskal-Wallis test, followed by a post-hoc Mann-Whitney U test to compare the sampling sites on each measured variable (i.e. abundance, holdfast area and blade length).

#### 2.3.2. PCA for morphological features and heavy metal concentrations

To examine data variability, a principal component analysis (PCA) was carried out [55] using abundance data, morphological features, and HMs concentrations among the sampling sites.

#### 2.3.3. Effect of seawater on spore release, settlement, gametophyte development, sexual differentiation, sporophyte production and fertility

Due to the distribution of the data, for all the *in vitro* experiments we performed a Kruskal-Wallis test (K-W), followed by a posteriori Mann-Whitney pairwise test to compare the treatments. For spore release, treatments were compared in terms of the accumulated number of spores released after 1, 2, 3, 5 and 7 h, each hour being analysed independently. For spore settlement, treatments were compared at 48 and 120 h after the beginning of the cultures. For gametophyte development, we performed two statistical analyses, (i) independently, a K-W on the percentage per day of each development stage (S%, G%, U%) and (ii) a two-way ANCOVA on all the recorded times per stage between the treatments. For sexual differentiation we compared the percentage of U, FG and MG after 4, 10 and 25 days of exposure between the treatments, also Dunn’s test was performed to compare the percentage per stage per treatment during the different exposure time. We compared the sporophyte production (%) and fertility (%) at 4, 10 and 25 days of exposure to the treatments. Finally, to evidence variation along the experimental time in these two descriptors, we performed another K-W test between the recorded times on each treatment.

## 3. Results

### 3.1. *In situ* analysis

#### 3.1.1. Abundance and morphometric features

**Fig 2:**
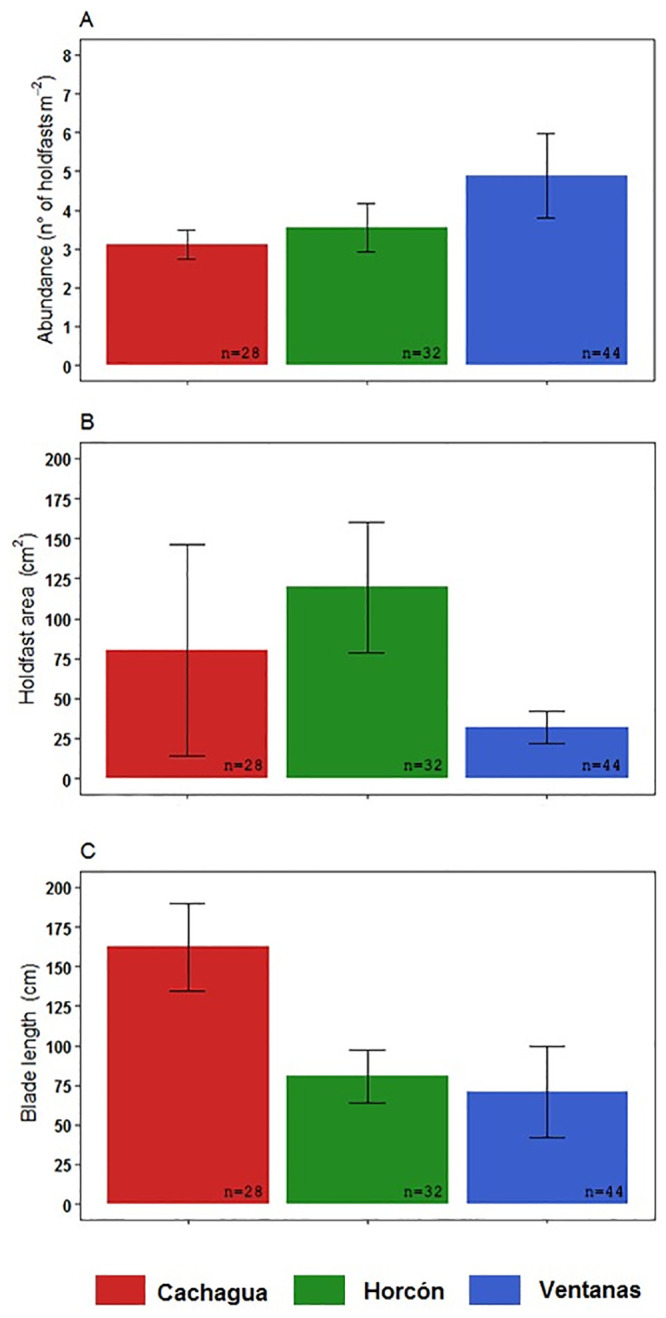
Comparison of morphological features. Average of A) abundance, B) holdfast area, and C) length of the longest blade of *Lessonia spicata* population from Cachagua, Horcón and Ventanas.

**Fig 3:**
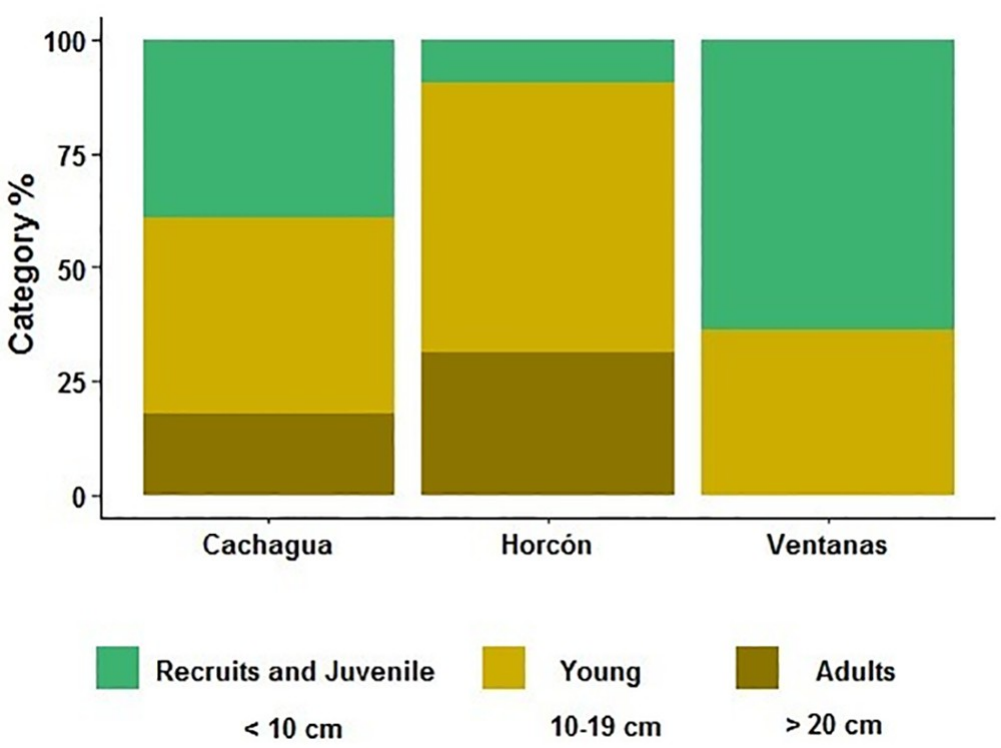
Comparison of size structure based on holdfast diameter. Percentual presence of three categories: recruit and juvenile, young, and adult sporophyte of *Lessonia spicata* populations. Each bar corresponds to one of the locations in this study: Cachagua, Horcón and Ventanas.

The K-W analysis revealed significant differences in abundance and morphometric traits between *L*. *spicata* populations from the different sampling sites. Abundances were significantly different among Ventanas and Cachagua (K-W = 0.028). Ventanas had the highest abundance (4.8 ± 2.4 holdfast m^-2^) in comparison with Horcón (3.5 ± 1.4 holdfast m^-2^) and Cachagua (3.1 ± 0.8 holdfast m^-2^) (Fig 2A). The holdfast area of *L*. *spicata* from Ventanas was also significantly different to Cachagua (K-W = 0.029), where Ventanas showed the smallest holdfast area (32 ± 9.9 cm^2^) in comparison with this site (80 ± 26.1 cm^2^) and Horcón (120 ± 40.5 cm^2^) (Fig 2B). Similarly, the length of blades was significantly different between Ventanas and Cachagua (K-W = 0.029), and between Horcón and Cachagua (K-W = 0.029) (Fig 2C). Ventanas displayed the shortest blades (71 ± 28.7 cm), in comparison to the intermediate blades from Horcón (81 ± 16.4 cm), and the longest blades from Cachagua (163 ± 27.6 cm). In relation to the size structure of *L*. *spicata* population, Cachagua showed 39.3% of recruits and juvenile plants, 42.9% of young and 17.9% of adult sporophytes. Horcón registered 9.4%, 59.4% and 31.3%, respectively (Fig 3). Finally, Ventanas presented only 63.6% of recruits and juvenile, 36.4% of young sporophytes and 0% of adult sporophytes (Fig 3).

#### 3.1.2. Heavy metal concentration in seawater and marine sediments

Regarding seawater from the three sampling sites, Cu and As occurred at maximal concentrations exceeding international normative for aquatic life by several orders of magnitude (Table 1). The Cu maximum values registered at each site, ordered from highest to lowest, were as follows: Cachagua (859 μg L^-1^), Ventanas (741 μg L^-1^) and Horcón (46 μg L^-1^). These values exceeded the EPA criteria for acute exposure (4.9 μg L^-1^) by 175 times in Cachagua, 151 times in Ventanas and 9.4 times in Horcón. In the case of As, the highest value was registered in Cachagua (1,484 μg L^-1^), followed by Horcón (449 μg L^-1^) and Ventanas (348 μg L^-1^). All three exceeded the EPA standard value for acute exposure (69 μg L^-1^) by 22, 7 and 5 times, respectively. On the other hand, Cd, Ag and Pb presented lower values than those recommended by international standards (Table 1).

**Table 1:** Range of concentrations of heavy metals in seawater from Cachagua, Horcón and Ventanas (2018).

	Range of concentration (μg L^-1^)				
Site	Cu	As	Ag	Cd	Pb
Cachagua	**19–859**	64–**1,484**	0.111–0.143	0.162–0.196	0.233–0.234
Horcón	**20–46**	6–**449**	0.057–0.207	0.065–0.335	0.363–2.261
Ventanas	**28–741**	9–**348**	0.010–1.191	0.091–0.243	0.093–3.425
acute	4.9	69	33	1.9	210
chronic	3.1	36	79	2	8.1

In marine sediments, Cu and As concentrations exceeded international standards by several orders of magnitude (Table 2). Specifically, the maximum Cu concentration was registered in Horcón (82,203 μg kg^-1^), followed by Ventanas (32,128 μg kg^-1^), both exceeding the CCME value for TEL (18,700 μg kg^-1^) by 4 and 2 times, respectively. In the case of As, the concentrations exceeded the CCME value for TEL (7,240 μg kg^-1^) by 3 times in Cachagua (20,895 μg L^-1^), 2 times in Horcón (14,008 μg L^-1^) and 1.8 times in Ventanas (13,743 μg L^-1^). On the contrary, Cd, Ag and Pb in the sediments showed lower values than those recommended by international criteria (Table 2).

**Table 2:** Range of concentrations of heavy metals in marine sediments from Cachagua, Horcón and Ventanas (2018).

	Range of concentration (μg Kg^-1^)				
Site	Cu	As	Ag	Cd	Pb
Cachagua	8,256–9,135	**20,271–20,895**	19–29	25–31	2,502–3,651
Horcón	**45,070–82,203**	994–**14,008**	28–66	20–80	2,115–3,109
Ventanas	5,285–**32,128**	776–**13,743**	10–70	17–49	1,310–3,709
TEL	18,700	7,240	700	-	30,200
PEL	108,000	41,600	4,200	-	112,000

These values correspond to the heavy metal concentrations in seawater (μg L^-1^) determined by ICP-MS. In bold are indicated values which exceeds the international norm [51] for acute (less than 24 h) and chronic exposure (more than 24 h).

These values correspond to the heavy metal concentrations in surface marine sediments (μg Kg^-1^) determined by ICP-MS. In bold are indicated values which exceeds the international norm [52, 53] for threshold effect levels (TEL) and Probable effect levels (PEL).

#### 3.1.3. Relationship between morphometric features and heavy metal concentrations

**Fig 4:**
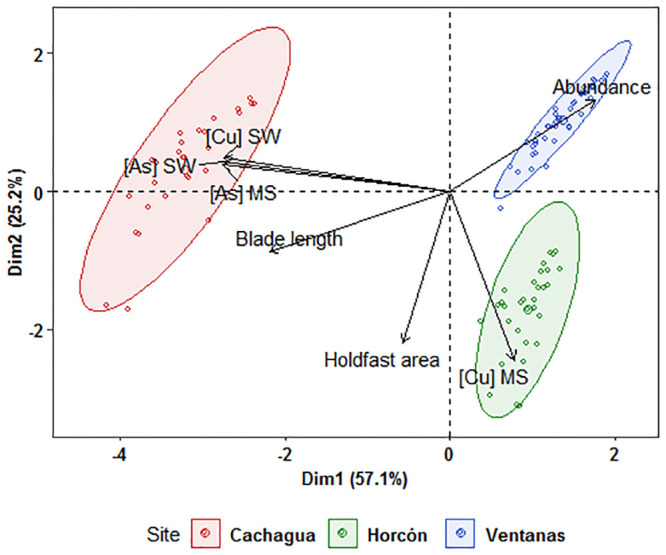
Principal components analysis of morphological features and heavy metals concentration. PCA includes abundance, holdfast area and blade length (Fig 2), plus the concentration of [As] and [Cu] in seawater (SW) and marine sediments (MS) (Tables 1 and 2).

Results of the PCA based on morphological characters and HMs concentrations are shown in Fig 4. The cumulative proportion of the first two principal components (Dim1 = 57.1% and Dim2 = 25.2%) explained more than 80% of the total variation in the data. This analysis evidenced three discrete kelp population clusters in relation to morphology, abundance, and concentrations of HMs in seawater and sediments (Fig 4). The most important morphological trait that allowed Cachagua data to differentiate was blade length, followed by kelp abundance (Fig 4). Furthermore, the most important environmental factors that allowed to separate Cachagua’s kelps population from the others in Dim1 were As and Cu concentrations in seawater and As in marine sediment (Tables 1 and 2).

### 3.2. *In vitro* experiments

#### 3.2.1. Effect of treatments on spore release

**Fig 5:**
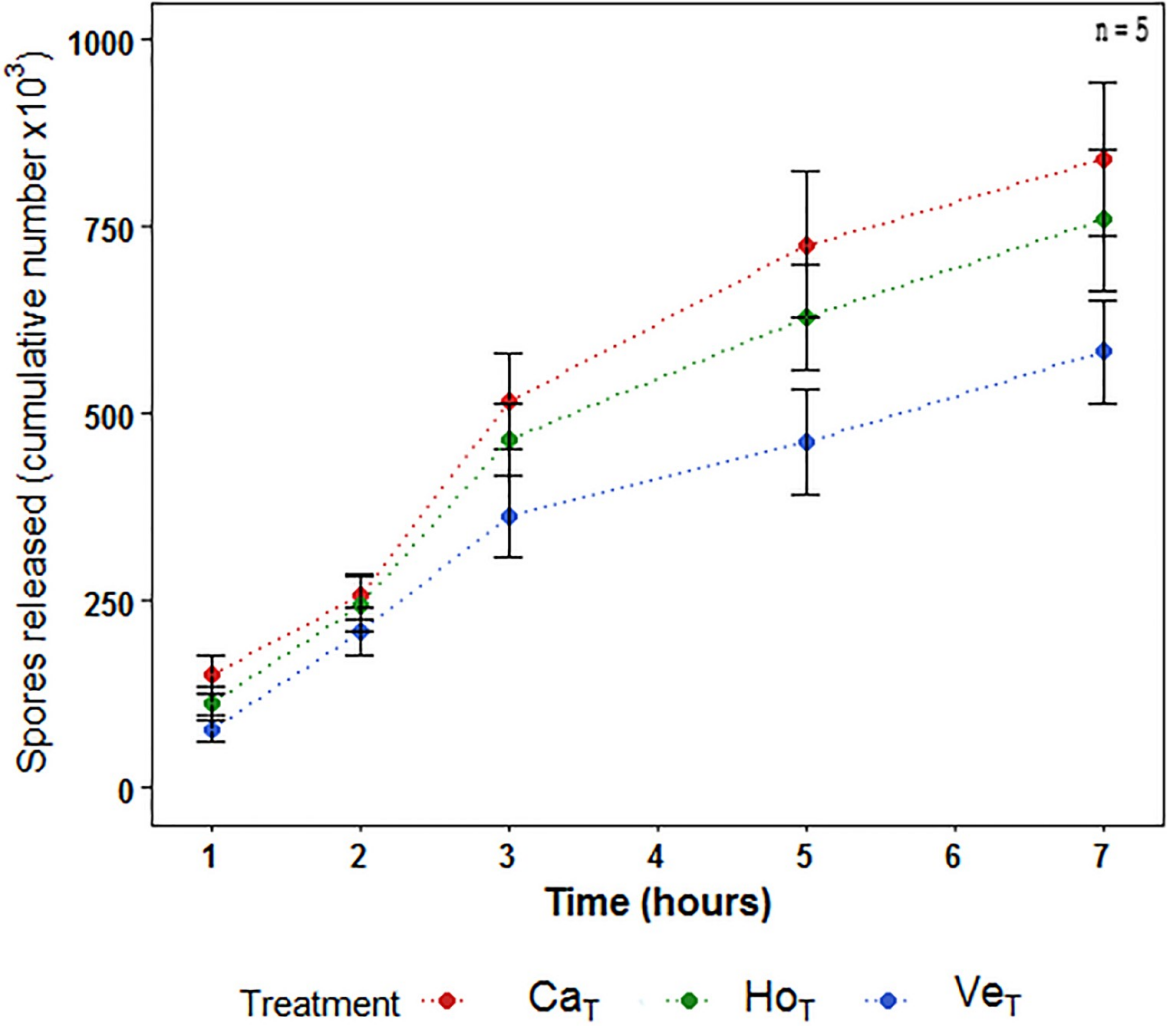
Spore released. Accumulated spores released by sori of *Lessonia spicata* (number of spores released mL^-1^, average ± S.D (n = 5). Ca; Cachagua, Ho; Horcón, Ve; Ventanas. T; treatment.

After three hours of exposure, spore release was higher using seawater from Horcón and Cachagua than with seawater from Ventanas (Fig 5). This tendency persisted until the end of the experiments (7 h) (spore release in Cachagua 841 ± 229 x 10^3^, Horcón 760 ± 212 x 10^3^ and Ventanas 583 ± 156 x 10^3^ spore mL^-1^). Significant differences were registered in the concentration of spores released between Cachagua and Ventanas (K-W = 0.042).

#### 3.2.2. Effect of treatments on spore settlement

**Fig 6:**
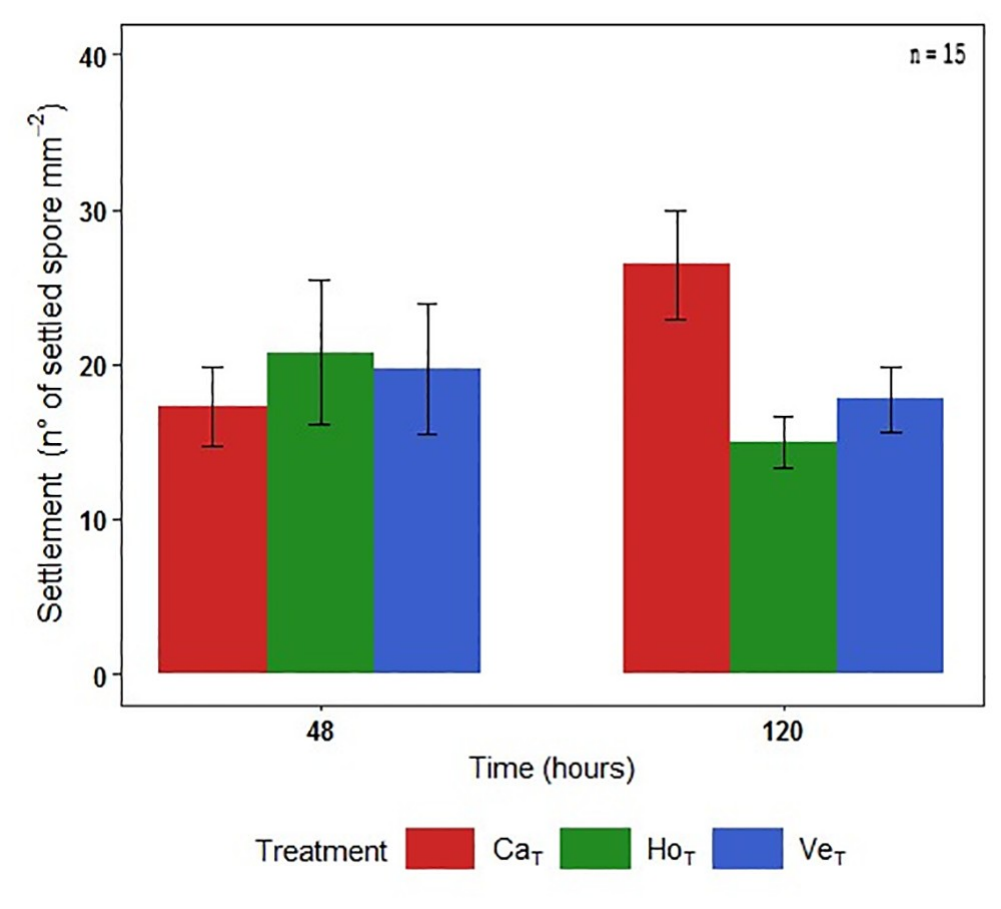
Settlement. Number of settled spores of *Lessonia spicata* per treatment showing SD (n = 15), at 48 and 120 h of exposure.

From 48 h to 120 h of exposure to the treatments, the number of settled spores in Horcón and Ventanas decreased 25%, while Cachagua showed an increase of 53% (Fig 6). Significant differences were registered between Cachagua and Horcón at 120 h of exposure (K-W = 0.014).

#### 3.2.3. Effect of treatments on gametophyte development

**Fig 7:**
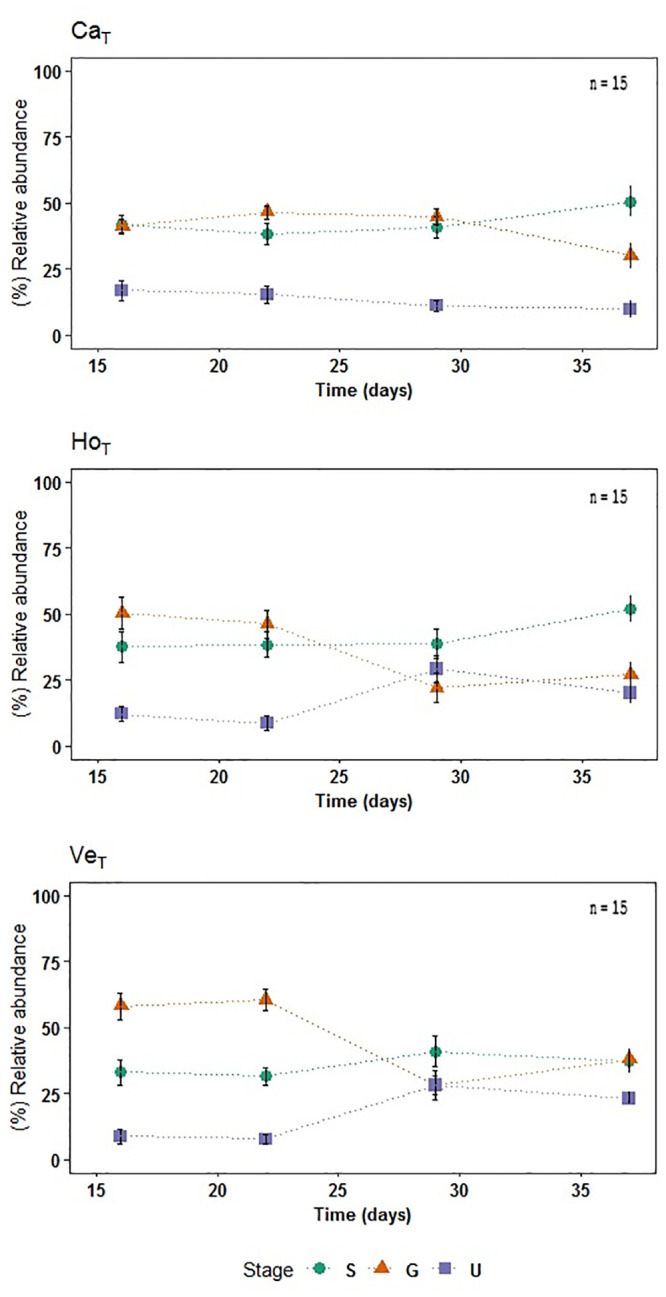
Gametophyte development. Development from settled spores to undifferentiated gametophyte during 37 days of exposure to the treatments, showing SD (n = 15). The legend indicates the following life cycle stages: Spores (S), Spores with germ tube (G) and Undifferentiated gametophyte (U) (Fig 3A–3C). Ca; Cachagua, Ho; Horcón, Ve; Ventanas. T; treatment.

The K-W test showed significant differences between treatments, and post hoc analyses showed that these differences were mainly between Ventanas and Horcón in comparison to Cachagua (S1 Table). The treatments Cachagua and Horcón had a higher percentage of undifferentiated gametophytes at day-16 (17% and 12%, respectively) in comparison with Ventanas (9%) (Fig 7), but K-W test did not show significant differences. Also, on day-25-30 of exposure, Horcón and Ventanas increased significantly (K-W = 0.003) their percentage of undifferentiated gametophytes (29% and 28%) in comparison to Cachagua (11%) (Fig 7). However, to compare if there were significant differences between the treatments over time, in relation to the percentage of each stage, a two ways ANCOVA was performed independently by stage. These results, evidenced that, the percentage of germinated spores (F = 0.002) and undifferentiated gametophytes (F = 0.0003) differed significantly between the treatments, where Ventanas had a higher percentage of germinated spores during a longer period compared with Cachagua. It is worth mentioning that the three treatments evidenced a high percentage of spores (35 ~ 46%) that did not develop during the entire exposure period (Fig 7).

#### 3.2.4. Effect of treatments on sexual differentiation

**Fig 8:**
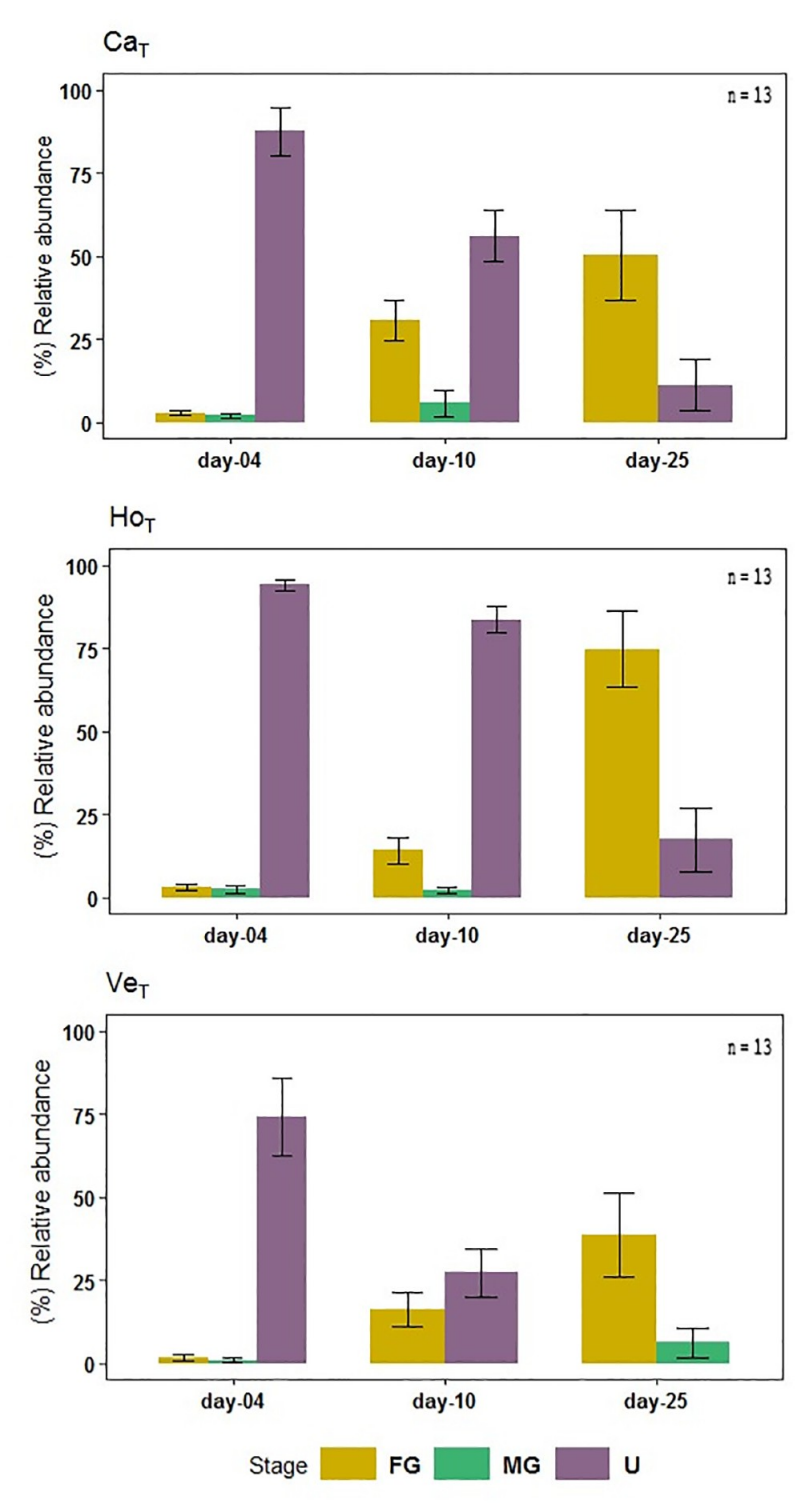
Sexual differentiation. Development from undifferentiated gametophyte to sexual gametophytes during 25 days of exposure to treatment, showing SD (n = 13). The legend indicates the different stages: Undifferentiated gametophyte (U), Female gametophyte (FG) and Male gametophyte (MG) (S2C–S2E Fig). Ca; Cachagua, Ho; Horcón, Ve; Ventanas. T; treatment.

At the beginning of the exposure, Cachagua and Horcón showed a small percentage of Female gametophyte (FG) (3%), which increased significantly to 50% and 74%, respectively (K-W = Cachagua = 0.027, Horcón < 0.001) over time (Fig 8). In Ventanas treatment, FG just increased from 2% to 39% (K-W = 0.024) (S2 Table). Also, undifferentiated gametophytes (U) remained high at Cachagua and Horcón at days 10 and 25 in comparison to Ventanas (K-W = Cachagua < 0.001, Horcón < 0.001) (Fig 8). A low percentage of Male gametophytes (MG) was registered in all treatments (Fig 8). Indeed, after 10 days of exposure the MG % in all treatment was lower than 10% (Cachagua = 6%, Horcón = 2% and Ventanas = 0%), and at the end of the experiment only Ventanas presented a 6% of MG (Fig 8).

#### 3.2.5. Effect of treatments on fertility and sporophyte production

**Fig 9:**
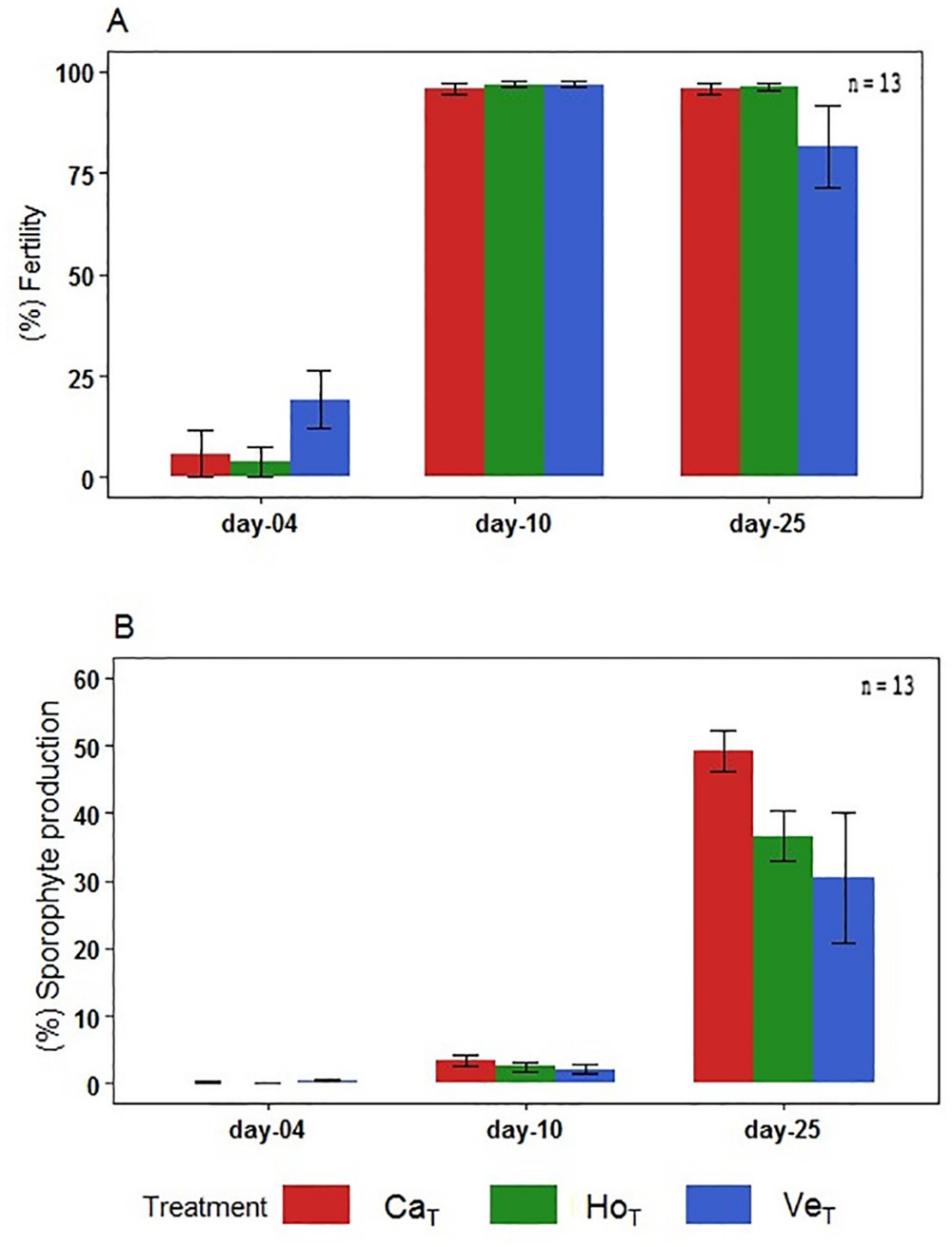
Fertility and sporophyte formation. A) Trends in the percentage of fertility and sporophyte formation based on the number of female gametophytes, and B) percentage of sporophyte production. Ca; Cachagua, Ho; Horcón, Ve; Ventanas. T; treatment.

Between days 4 and 10 of exposure, the three treatments displayed an increase of fertility, corresponding to approximately 96–97% (Fig 9A). Between days 10 and 25, this percentage remained stable in Cachagua and Horcón, but fertility in Ventanas decreased to 81% (Fig 9A). On the other hand, the sporophyte production indicated that Cachagua and Horcón significantly increased their percentage of sporophytes from 0 to 49% and from 0% to 37%, respectively (K-W = Cachagua < 0.001, Horcón < 0.001) (Fig 9B). Ventanas was the treatment with the lowest percentage of sporophyte production (31%) and showed significant differences in comparison to the other sites (K-W = 0.035).

## 4. Discussion

There are many factors involved in the fitness and development of seaweeds such as *L*. *spicata*, especially in terms of pollution impact evaluations. One unexpected result of this work is that the HMs concentration of Cu and As in seawater and sediment samples was above the international normative in all three sampling sites. Additionally, our results supply information that suggests that both adults and early life stages of this kelp are negatively affected once exposed to seawater from sites that are closer to a highly polluted focus, such as the industrial park of Quintero, rather than distant sites. Specifically, in terms of morphological features, all the sampling sites of this study presented average densities of *L*. *spicata* that were considerably lower (< 5 holdfast m^-2^) than those previously reported in the coast of Central Chile [56, 57]. Additionally, for this species, it is reported that the maximum fertility is observed in July [47]. However, the site that is closest to the pollution source (Ventanas), had the largest percentage of recruits and juveniles (62%) and fully mature adults were absent. Indeed, the adult kelps from Ventanas exhibited the smallest average holdfast area (32 cm^2^), the shortest blades (71 cm) and width diameter (8.7 cm), in comparison with the other two sites. On the contrary, *L*. *spicata* individuals from the most distant site (Cachagua) showed a higher heterogeneity in terms of morphology and showed the highest average length of blades, implying that this population presented both, young and old individuals. Also, Horcón was slightly separated from Ventanas in the PCA, because it showed the highest concentrations of Cu in marine sediments and the larger kelp average holdfast area, representing a transition zone in terms of morphology. However, the three kelp populations were clearly separated in relation to their morphometry and heavy metal concentrations in seawater and sediments (Fig 4). These results were similar to those reported by Vásquez et al. [58] who found a negative relationship between the size of adult individuals of the kelp *Lessonia trabeculata* and proximity to a mining pollution source. More specifically, these authors observed that populations of *L*. *trabeculata* were absent in the first 0 to 3 km from the pollution source, whereas from 3 to 5 km kelp individuals were present, and their sizes increased significantly with distance. These results are related with the evidence that heavy metals excess in algae triggers an overproduction of reactive oxygen species (ROS) and leads to an oxidative stress condition, which negatively affects kelps ultrastructure, growth, and physiology [4, 58]. Indeed, a possible explanation for the variation of morphological features among the populations in this study is based on Sáez et al. [16], who demonstrated in *L*. *trabeculata* that patterns of metal accumulation in kelps parts such as holdfast, stipe and blades are metal specific and each part is impacted differently.

In terms of HMs concentration, all three sites were highly polluted with Cu and As above the international normative; nonetheless, Ag, Cd and Pb concentrations in sediment samples of the three sites were at least one order of magnitude inferior to those previously reported for QB by Parra et al. [41]. The historical environmental information about Ventanas suggests that other pollutants may be present in seawater, such as polycyclic aromatic hydrocarbons (PAHs) resulting from oil refinery activities (in higher concentrations than in Horcón and Cachagua) and could explain the smaller kelp sizes from Ventanas. We did not find specific information about population adaptations to chemicals such as PAHs in seaweeds, but it has been previously demonstrated that photosynthetic organisms under sublethal stresses (e.g. chemicals, temperature) incur a fitness cost, reallocating resources to costly stress responses and maintenance of homeostasis [59]. Accordingly, Ventanas kelp individuals would have been affected in a similar way as terrestrial plants, where physico-chemical stressors result in growth inhibition, decreased biomass and smaller body sizes, plus impacts on other traits that we did not asses such as presence of reproductive tissue or photosynthetic activity [60]. So, based on our results plus the environmental history of Ventanas, we suggest that adult kelps of this site reached shorter sizes than those from less-polluted environments due to excess of different pollutants and to a long-term exposure, i.e. chronic exposure to pollutants.

The analysis of seawater and marine sediments samples showed that our initial premise was wrong because the pollution did not decrease significantly with increasing distance from the industrial focus. In fact, unexpectedly, in relation to Cu and As levels in seawater and sediment samples, all three sites showed levels above the international norm (EPA, [51]) for acute and chronic exposure in seawater, and for TEL and PEL in sediments (according to the CCME, [53]) (Tables 1 and 2). The only exception to this trend was the relatively unpolluted sediments of Cachagua for Cu concentration. Indeed, seawater samples from Ventanas and Horcón exhibited high Cu concentrations similar to those previously reported by Contreras [30] and FIC [36] in QB. Nevertheless, among the three sites, Cachagua showed the highest concentrations of Cu in seawater, and also the highest levels of As in sediments. Regarding Cu levels in sediments, Horcón registered the highest value among the three sites (82,203 μg kg^-1^). Horcón sediments probably showed this high Cu concentration because this sampling site is a fishing cove, where high concentrations of organic matter occur, which facilitates Cu sedimentation and increases the concentration of this metal in marine sediments [61].

In our study, maximum Cu concentrations in sediments were in the same order of magnitude as those reported within QB by De Gregori et al. [62], Sáez et al. [63] (52,000 and 68,000 μg kg^-1^, respectively) but lower than those of Parra et al. [41]. Specifically, Cachagua and Ventanas displayed maximum Cu concentrations in sediments that were lower compared to the control site reported by Parra et al. [41] (41,000 μg kg^-1^); on the other hand, Cu level at Horcón was higher than the control but lower than most of the sampling stations of Parra et al. [41] (range 41,000–1,476,000 μg kg^-1^). Moreover, sediment samples from Horcón and Ventanas showed maximum As concentrations that were similar to those reported within QB by Parra et al. [41]; on the contrary, Cachagua’s sediment samples exhibited a maximum As concentration that was as high as the most polluted sampling station of Parra et al. [41], which was located just in front of the main copper refinery at QB. The concentration of Cd, Ag and Pb in sediments was also lower than those reported for the sampling station used as control in a previous study of Parra et al. [41], where the concentrations correspond to samples collected closer to the industrial discharge in comparison to our study, i.e. mostly within QB.

In summary, for sediment samples, a mixed result was observed. On the other hand, Cu and As levels in the seawater from all three sites were above the international norm and were higher than those of previous reports for more distant sites such as Cachagua. These results agree with the findings of Celis et al. [64], who reported higher concentrations of HMs (As, Pb and Cu) in Humboldt penguin feces (*Spheniscus humboldti*) from Cachagua Island compared to previous reports. In the past, this site was indeed considered a non-impacted site, with reported Cu concentration values of < 1 to 5 μg L^-1^ in seawater [13, 41]. One possible explanation for the high concentrations of As and Cu found in Cachagua could be the intensification of industrial activity in the Valparaíso region without specific normative for the reduction of pollution in this zone, and the increase and expansion of environmental pollution from QB [65]. Because the water currents along the Humboldt Current System (HCS) flow predominantly northward [66], we can hypothesize that in Cachagua (located north of the industrial focus of QB), the HCS would provide a constant and frequent flux of polluted water masses and/or polluted biomass, carrying high concentrations of HMs. Consequently, this work supplies more evidence to suggest a pollution expansion from Quintero Bay along the Chilean coast.

The results from our *in vitro* experiments showed different responses depending on the developmental process or stage. However, the early life stages of *L*. *spicata* exposed to Cachagua seawater exhibited a greater performance than when they were exposed to seawaters from the two sites closest to the pollution source of QB. Although the concentration of total Cu and As in the seawater of Cachagua was higher in comparison with the other two sites (see previous section), our results indicate a tendency of higher spore release and spore settlement in the Cachagua treatment than in the Ventanas and Horcón. Spore release of Cachagua was approximately 30% higher than Ventanas, but only 10% higher than Horcón treatment at 7 h of exposure (Fig 5). Similarly, the reduction of spore settlement in both Ventanas and Horcón treatments compared to Cachagua was 25–30% after 120 h of exposure (Fig 6). Because, as previously mentioned, seawater samples collected from all three sites displayed Cu and As levels above the international criteria, it is not likely that these two compounds explain this differential performance. Thus, we hypothesize that additional pollutants (e.g. PAHs) which are present in the seawater from Ventanas and Horcón, and which are probably present at lower concentration in Cachagua’s seawater, might significantly enhance the effects of HMs (synergic effects). In fact, previous studies have demonstrated the presence of several PAHs in the seawater of Ventanas and Horcón, such as: acenaphthene, benzo (a) pyrene, phenanthrene, pyrene, among others [36]. Additionally, human activities such as the burning of fossil fuels, coal and biomass, or ship transport and oils spills have been shown to occur frequently in both sites, all causing a significant rise in PAHs levels [36, 67]. Indeed, Contreras-Porcia et al. [17] recently evidenced synergistic negative effects of binary mixtures of PAHs and HMs (Cu and Cd) on spore release and settlement in *M*. *pyrifera* and *L*. *spicata*; probably explaining the lower performance of Ventanas and Horcón treatments compared to Cachagua. On the other hand, Contreras et al. [18] showed that the exposure of *L*. *berteroana* to Cu concentrations equal or higher than 8 μg L ^-1^ cause a decrease (over 50%) on spore release. This would indicate that populations of *L*. *spicata* that are closer to the QB industrial park in Central Chile have developed a greater tolerance to Cu than *L*. *berteroana*, and probably other populations of *L*. *spicata* from less impacted sites.

Spore germination and gametophyte development of *L*. *spicata* was achieved successfully, with the presence of undifferentiated gametophytes in all three seawater treatments after 13 days of culture, which is within the usual time frame previously reported for this species under similar culture conditions. Although the number of undifferentiated gametophytes was higher in Cachagua’s treatment than in the others, and some delay in gametophyte development could be suggested in Ventanas and Horcón treatments compared to Cachagua (see Results), no major developmental differences were evident from data analyses. Nonetheless, all three treatments showed a large proportion of spores that did not germinated, and of germinated spores that did not develop into gametophytes, during the 37 days of the culture period (Fig 7). A previous study on the kinetics of early life stage development of *L*. *spicata* from central Chile showed that, under no addition of toxic (control condition), one third of spores did not germinate during forty days of culture. Nonetheless, for the rest of more advanced early life stages, this last study showed that each successive life stage (germinated spores, undifferentiated and differentiated gametophytes, and sporophytes) followed a phase of growth and then a sharp decrease in their numbers until its development (with some time delay) to pass to the next life stage. Contreras et al. [18] also demonstrated that when treated with increasing amounts of Cu, as in our case, a large proportion of spores of *L*. *spicata* do not germinate, and a large proportion of germinated spores do not develop into undifferentiated gametophytes; moreover, very high concentrations of Cu resulted in a complete inhibition of post-germination stages, which was not the case for our three seawater treatments (see Fig 6 of Contreras et al. [18]). This supports the fact that, probably, although the seawater from all three sites considered in the present study had high levels of pollutants, early life stages of *L*. *spicata* from central Chile were able to cope with these high levels of pollution, even though they undergo sub-lethal effects (such as a delayed development). Undifferentiated gametophytes were observed during the 40 days of exposure in all treatments, lasting longer than the documented normal period of development under standard culture conditions [68]. This is in accordance with the findings by Bond et al. [69] who demonstrated a delay in the development of germlings of *Fucus spiralis* when exposed to an excess of Cu. Similarly, our results agree with Chung & Brinkhuis [70], who found that Cu concentrations greater than or equal to 50 μg L^-1^ caused a delay in gametophyte development of *Laminaria saccharina*. On the other hand, Tala et al. [71] reported the recovery capacity of early life stages of *Lessonia* species after stress, evidencing that surviving spores retained their viability.

Our results showed that under exposure to the three seawater treatments, sexual differentiation of gametophytes was attained during the culture period (at day 25) despite the high HMs concentration used. Although percentages of differentiated gametophytes were lower for Ventanas than Cachagua and Horcón (Fig 8), the proportion of male (5%) to female gametophytes (95%) was extremely female-biased (sex ratio = 0.05) in all treatments. Previous studies have demonstrated that in natural populations of *L*. *spicata*, male and female gametophytes generally occur in equal proportions (sex ratio = 0.5) [72]. However, this ratio could change under T°, pH or other environmental fluctuations [20, 72, 73]. For example, Leal et al. [20] evidenced the interactive effects of pH, T° and Cu on the sex ratio of *M*. *pyrifera* and *Undaria pinnatifida*. A decrease in the proportion of male to female gametophytes has also been previously reported for the Chilean coast. For example, Oppliger et al. [74] determined that in *L*. *spicata* populations from cooler southern sampling sites (El Quisco, Las Cruces and Valdivia), the proportion of male gametophytes decreased significantly with temperature, compared to the warmer northern sites. Likewise, Nelson [75] reported that cultivation of *Lessonia variegata* under the combined conditions of 15°C and a photoperiod of 15:9 results in a female-biased sex ratio, suggesting that males are less tolerant to stressful conditions than females. In this context, and according to our results and previous literature, we postulate that male gametophytes of *L*. *spicata* are more sensitive to polluted seawater than female gametophytes. Moreover, since all three treatments considered in this study exhibited an extremely female-biased sex ratio, this would indicate that *L*. *spicata* populations from the three sites are probably subjected to sublethal toxic effects resulting from high levels of pollution. All three treatment cultures showed a considerable increase in fertility after 4 days of culture (80–90%) (Fig 9), although the number of male gametophytes formed was extremely low. One possible explanation of this sex ratio bias could be parthenogenesis. Parthenogenesis, frequently involving the development of a female gamete without male fertilization, has been reported in terrestrial and marine organisms, and is a form of asexual reproduction which is prevalent in stressful environmental conditions, in marginal populations [74]. Parthenogenesis can modify the sex ratio, and it has already been reported to occur in *Lessonia* species in nature, as well as under controlled culture conditions [54, 74]. Additionally, we determined that Ventanas was the treatment with the lowest percentage of sporophyte production (31%) which showed significant differences compared to the other two treatments. However, the sporophytes production (25–50%) in all the treatments did not increase significantly over exposure time, indicating sub-lethal toxic effect of pollution on *L*. *spicata* sporophyte production. A disruption of sporophyte development was also previously demonstrated in *Laminaria hyperborea* chronically exposed to Cu excess, which would affect population’s stability in HMs impacted sites [76]. Therefore, our results suggest that chemical pollution has a significant impact on sporophyte production in *L*. *spicata* populations from sites that are highly impacted by anthropogenic pollution. Moreover, parthenogenesis, which would result from a low viability of male gametophytes under chemical stress conditions, could be operating in these polluted environments, probably explaining the extremely female-biased sex ratio.

Some previous studies demonstrated [77, 78] that there is an interactive effect of *in situ* environmental conditions (such as temperature, day length, light intensity, and ultraviolet radiation) on gametophyte and sporophyte growth and survival. This would explain the temporal (stationary) variation in the gametophyte and sporophyte performance in *Ecklonia radiata* and *Laminaria solidungula*, respectively. Our *in vitro* experiments were performed using sori collected from a single site belonging to the same *L*. *spicata* population and were additionally subjected to the same laboratory conditions. Therefore, we discard that different environmental conditions would significantly explain the differences in performance between early life stages of *L*. *spicata* treated with different seawater treatments. On the contrary, it is more likely that the differences observed in gametophyte development (and other early life stages) in our *in vitro* experiments would be related to the different pollutants and concentrations, presents in the three types of seawater used in toxic treatments.

It is important for future studies to highlight that the high concentrations of HMs in all three sampling sites, both near the industrial park of Quintero Bay and 40 km away, suggest an expansion of industrial pollution from QB since the year 2000. In fact, in all three *L*. *spicata* populations surveyed in this study, the density of adult individuals was significantly lower than previously reported in central Chile. The small sizes of kelps would have negative bottom-up effects on higher trophic levels [79]. In fact, kelps provide a three-dimensional habitat for biodiversity, which depends on their sizes and morphology. Specifically, Villegas et al. [80] showed that in the case of the kelp *L*. *trabeculata*, the maximum holdfast diameter is strongly and positively correlated with the size (and the weight) of other morphological traits; and that these morphological variables drive the invertebrate biodiversity. Although a tendency of higher performance of early life stages was observed in the treatment using seawater from Cachagua compared to Horcón and Ventanas, sublethal effects of pollution on early life stages (spore release, settlement, gametophyte and sporophyte production) were observed in all three treatments. This confirms that all three sites have differential but high levels of pollution, which contradicted our initial premise that Cachagua would be less polluted than the other two sites. Remarkably, relatively low sporophyte production and fertility, and an extremely female-biased sex ratio (0.05) were observed under seawater exposure from all three sites. This suggests that parthenogenesis could be operating under these chemically polluted and stressful conditions. In conclusion, this work supports that pollution expansion has already occurred along the central coast from Quintero Bay, negatively affecting the quality of seawater, sediments, kelp populations, and probably the entire marine ecosystem. All the previous results imply that there is an urgent need to improve the environmental normative determining the maximum levels of pollutants allowed, and the environmental pollution control programs and policies of Chile, especially considering foundation kelp species.

## Supporting information

S1 Fig Map of sampling sites. Georeferencing of the sampling sites, the framed area indicates the Industrial Park “Las Ventanas” located in Quintero Bay, Valparaíso Region. From north to south; Cachagua, Horcón and Ventanas.
(TIF)

S2 Fig Early developmental stages of *Lessonia spicata*. Inverted microscope photos of: A. Spore (S), B. Germinated spore (G), C. Undifferentiated gametophyte (U), D. Male gametophyte (MG) and E. Female gametophyte with sporophytes and an egg cell (arrow) (FG).
(TIF)

S3 Fig Scheme of *in vitro* experiments. Scheme about time of developmental stages and each data recording. The colour bars indicate the time of respective stage recording. In the case of spore release, settlement and germination and gametophyte development, the darker colour indicates the data time that was considered for the statistical analysis.
(TIF)

S1 Table Statistical analysis of gametophyte development. Results of Kruskal-Wallis and Mann-Whitney pairwise comparisons between treatments per day per stage during gametophyte development; Spore (S), Germinated spore (G) and Undifferentiated gametophyte (U). Different letters indicate statistical differences between the treatments.
(DOCX)

S2 Table Statistical analysis of sexual differentiation. Results of Kruskal—Wallis and Dunn’s test for each developmental stage percentage (Undifferentiated gametophyte (U), Female gametophyte (FG) and Male gametophyte (MG)) between 4, 10 and 25 days of exposure per treatment.
(DOCX)

## Abbreviations

Ca_T_: Treatment using seawater from Cachagua
CCME: Canadian Council of Ministers of the Environment
EPA: Environmental Protection Agency
FG: Female gametophyte
G: Germinated spore
HMs: Heavy metals
Ho_T_: Treatment using seawater from Horcón
MG: Male gametophyte
PAHs: Polycyclic aromatic hydrocarbons
PEL: Probable effect levels
QB: Quintero Bay
S: Spore
TEL: Threshold effect levels
U: Undifferentiated gametophyte
Ve_T_: Treatment using seawater from Ventanas
