# Supplementary figures and images for: Coastal pollution from the industrial park Quintero bay of central Chile: Effects on abundance, morphology, and development of the kelp Lessonia spicata (Phaeophyceae)
Source: PLoS One. 2020 Oct 15;15(10):e0240581. doi: 10.1371/journal.pone.0240581 (PMC7561192; doi:10.1371/journal.pone.0240581)

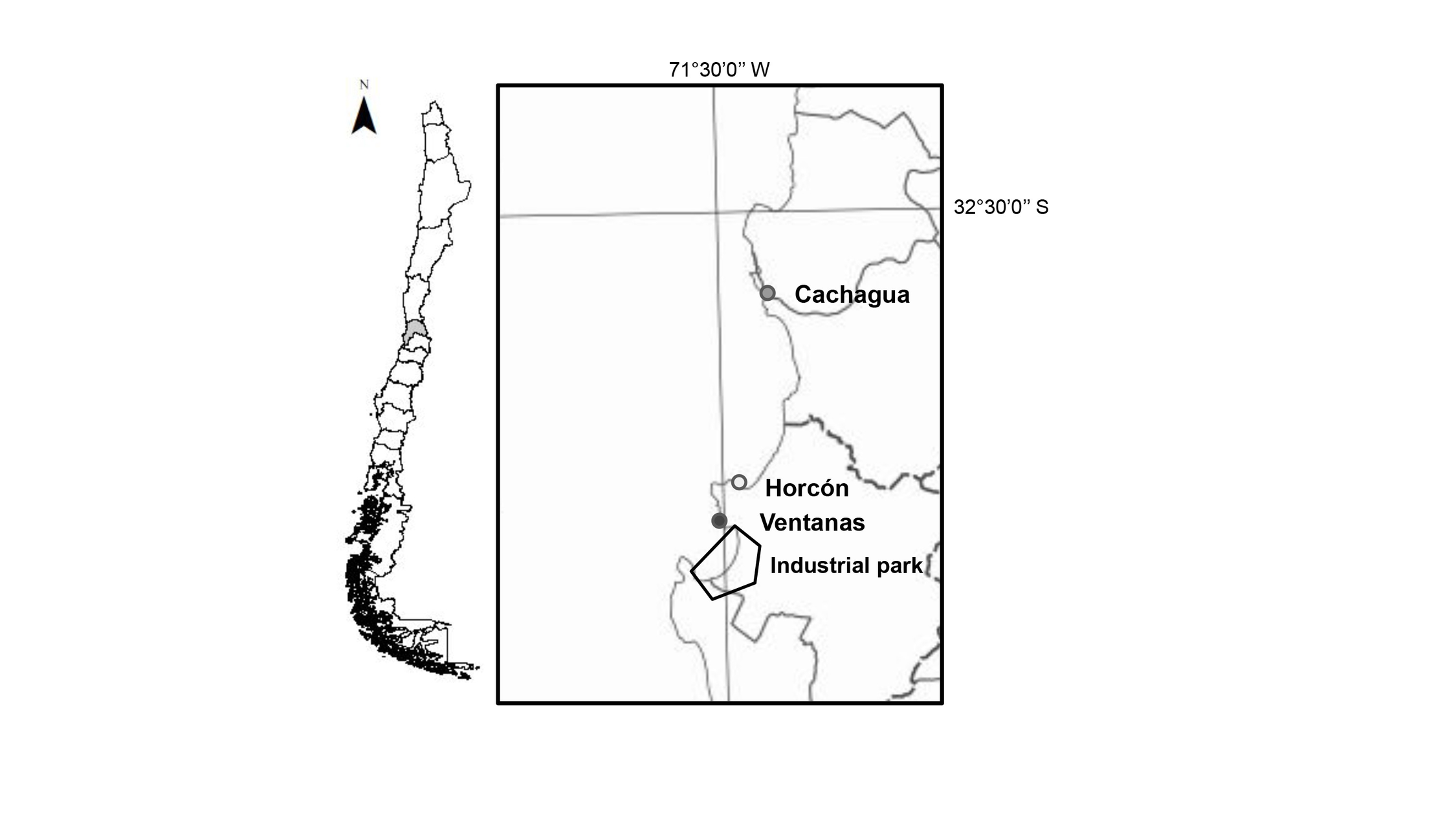

Supplement: S1 Fig — Georeferencing of the sampling sites, the framed area indicates the Industrial Park “Las Ventanas” located in Quintero Bay, Valparaíso Region. From north to south; Cachagua, Horcón and Ventanas. (TIF) [file pone.0240581.s001.tif]

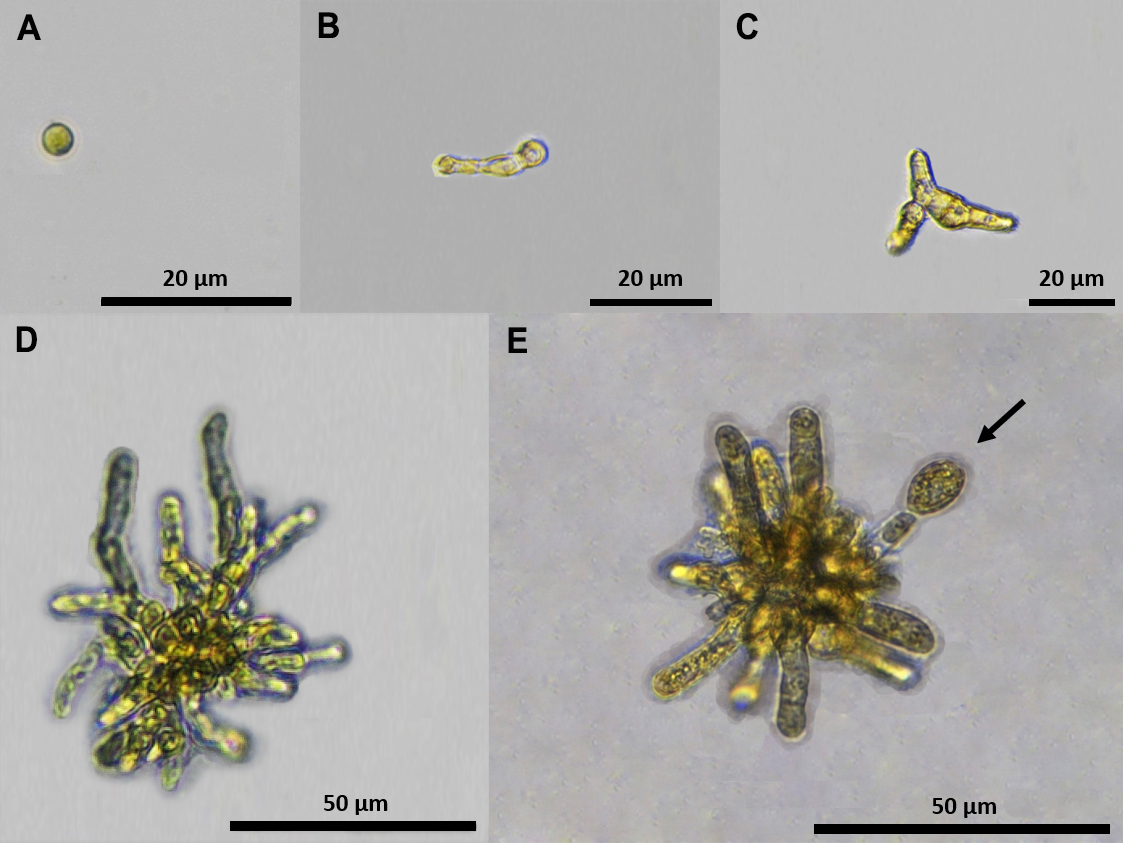

Supplement: S2 Fig — Inverted microscope photos of: A. Spore (S), B. Germinated spore (G), C. Undifferentiated gametophyte (U), D. Male gametophyte (MG) and E. Female gametophyte with sporophytes and an egg cell (arrow) (FG). (TIF) [file pone.0240581.s002.tif]

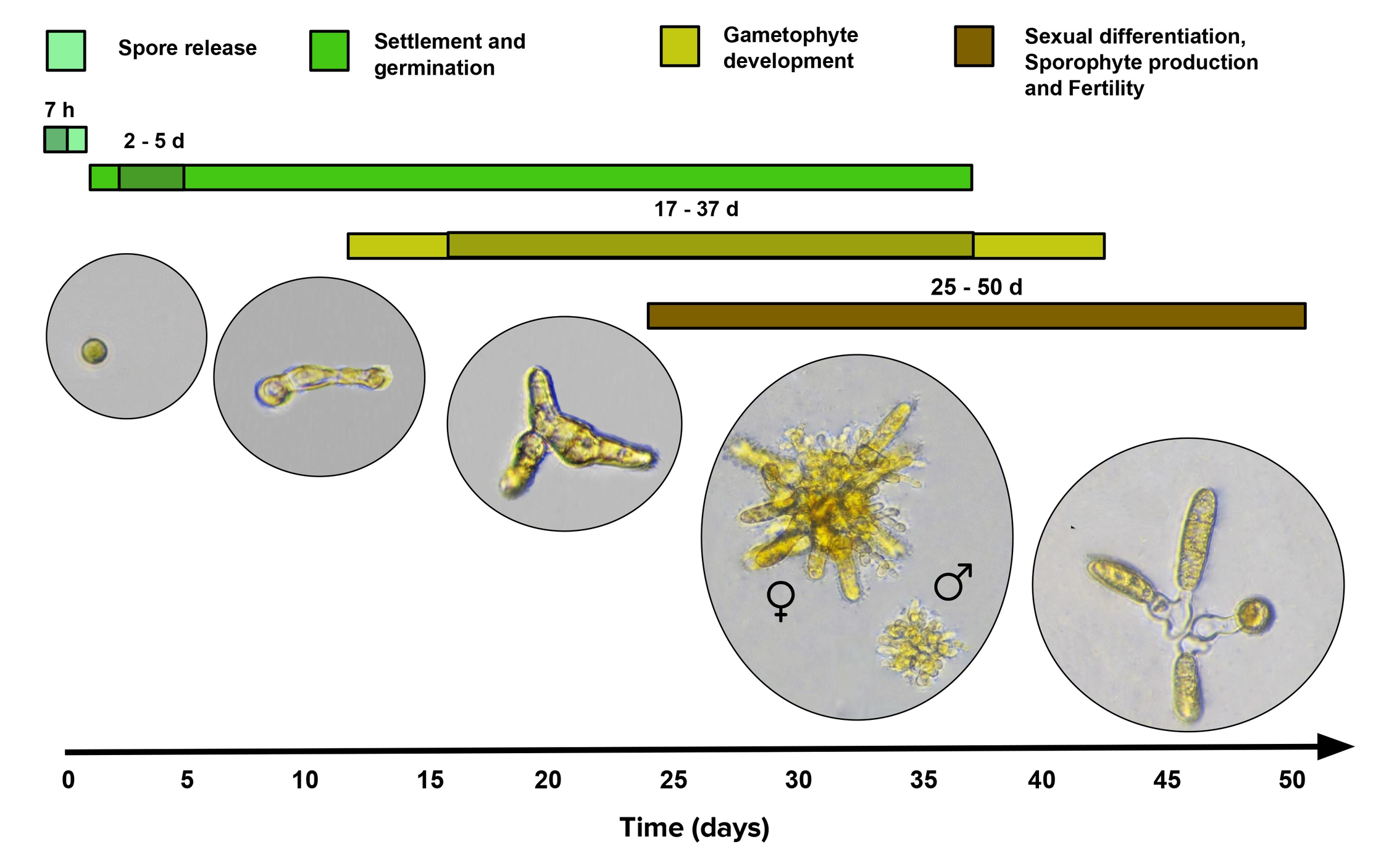

Supplement: S3 Fig — Scheme about time of developmental stages and each data recording. The colour bars indicate the time of respective stage recording. In the case of spore release, settlement and germination and gametophyte development, the darker colour indicates the data time that was considered for the statistical analysis. (TIF) [file pone.0240581.s003.tif]
