# Supplementary material for: Coastal pollution from the industrial park Quintero bay of central Chile: Effects on abundance, morphology, and development of the kelp Lessonia spicata (Phaeophyceae)
Source: PLoS One. 2020 Oct 15;15(10):e0240581. doi: 10.1371/journal.pone.0240581 (PMC7561192; doi:10.1371/journal.pone.0240581)
Supplement: S1 Table — Results of Kruskal-Wallis and Mann-Whitney pairwise comparisons between treatments per day per stage during gametophyte development; Spore (S), Germinated spore (G) and Undifferentiated gametophyte (U). Different letters indicate statistical differences between the treatments. (DOCX) [file pone.0240581.s004.docx]

**S1 Table.** Results of Kruskal - Wallis and Mann Whitney pairwise comparisons between treatments per day per stage during gametophyte development; Spore (S), Germinated spore (G) and Undifferentiated gametophyte (U). Different letters indicate statistical differences between the treatments.

| **Day** | **Stage** | Ca_T_ | Ho_T_ | Ve_T_ | **P-value** | **H(chi2)** |
| --- | --- | --- | --- | --- | --- | --- |
| **16** | **G** | b | ab | a | 0.027 | 7.144 |
| **16** | **U** | - | - | - | 0.233 | 2.834 |
| **22** | **S** | - | - | - | 0.321 | 2.266 |
| **22** | **G** | b | ab | a | 0.050 | 5.932 |
| **22** | **U** | - | - | - | 0.090 | 4.71 |
| **29** | **S** | - | - | - | 0.934 | 0.135 |
| **29** | **G** | a | b | b | 0.006 | 10.02 |
| **29** | **U** | b | a | a | 0.003 | 11.06 |
| **29** | **S** | - | - | - | 0.088 | 4.855 |
| **37** | **G** | - | - | - | 0.279 | 2.546 |
| **37** | **U** | b | ab | a | 0.017 | 7.961 |
