# Supplementary material for: Coastal pollution from the industrial park Quintero bay of central Chile: Effects on abundance, morphology, and development of the kelp Lessonia spicata (Phaeophyceae)
Source: PLoS One. 2020 Oct 15;15(10):e0240581. doi: 10.1371/journal.pone.0240581 (PMC7561192; doi:10.1371/journal.pone.0240581)
Supplement: S2 Table — Results of Kruskal—Wallis and Dunn’s test for each developmental stage percentage (Undifferentiated gametophyte (U), Female gametophyte (FG) and Male gametophyte (MG)) between 4, 10 and 25 days of exposure per treatment. (DOCX) [file pone.0240581.s005.docx]

**S2 Table.** Results of Kruskal - Wallis and Dunn’s test of percentage of each developmental stage (Undifferentiated gametophyte (U), Female gametophyte (FG) and Male gametophyte (MG)) between 4, 10 and 25 days of exposure per treatment.

| **Treatment** | **Stage** | **P value** | **H(chi2)** | **Dunn test**  **(Interaction 04 - 25 days)** |
| --- | --- | --- | --- | --- |
| Ca_T_ | **U** | <0.001 | 23.079 | <0.001 |
| Ca_T_ | **FG** | 0.027 | 7.173 | 0.029 |
| Ca_T_ | **MG** | 0.056 | 5.739 | 0.024 |
| Ho_T_ | **U** | <0.001 | 20.092 | <0.001 |
| Ho_T_ | **FG** | <0.001 | 15.933 | <0.001 |
| Ho_T_ | **MG** | 0.056 | 5.739 | 0.024 |
| Ve_T_ | **U** | <0.001 | 15.132 | <0.001 |
| Ve_T_ | **FG** | 0.062 | 5.556 | 0.027 |
| Ve_T_ | **MG** | 0.203 | 3.182 | 0.524 |
